# Towards a Miniaturized Photoacoustic Sensor for Transcutaneous CO_2_ Monitoring

**DOI:** 10.3390/s24020457

**Published:** 2024-01-11

**Authors:** Mahmoud El-Safoury, Christian Weber, Hassan Yassine, Jürgen Wöllenstein, Katrin Schmitt

**Affiliations:** 1 Fraunhofer Institute for Physical Measurement Techniques IPM, 79110 Freiburg im Breisgau, Germany; christian.weber@ipm.fraunhofer.de (C.W.); juergen.woellenstein@ipm.fraunhofer.de (J.W.); katrin.schmitt@ipm.fraunhofer.de (K.S.); 2 Department of Microsystems Engineering–Institut für Mikrosystemtechnik (IMTEK), University of Freiburg, 79110 Freiburg im Breisgau, Germany; hassan.yassine@imtek.uni-freiburg.de

**Keywords:** photoacoustic sensor, carbon dioxide (CO_2_), transcutaneous, light-emitting diode (LED), micro-electro-mechanical system (MEMS) microphone, two-chamber photoacoustic system

## Abstract

A photoacoustic sensor system (PAS) intended for carbon dioxide (CO_2_) blood gas detection is presented. The development focuses on a photoacoustic (PA) sensor based on the so-called two-chamber principle, i.e., comprising a measuring cell and a detection chamber. The aim is the reliable continuous monitoring of transcutaneous CO_2_ values, which is very important, for example, in intensive care unit patient monitoring. An infrared light-emitting diode (LED) with an emission peak wavelength at 4.3 µm was used as a light source. A micro-electro-mechanical system (MEMS) microphone and the target gas CO_2_ are inside a hermetically sealed detection chamber for selective target gas detection. Based on conducted simulations and measurement results in a laboratory setup, a miniaturized PA CO_2_ sensor with an absorption path length of 2.0 mm and a diameter of 3.0 mm was developed for the investigation of cross-sensitivities, detection limit, and signal stability and was compared to a commercial infrared CO_2_ sensor with a similar measurement range. The achieved detection limit of the presented PA CO_2_ sensor during laboratory tests is 1 vol. % CO_2_. Compared to the commercial sensor, our PA sensor showed less influences of humidity and oxygen on the detected signal and a faster response and recovery time. Finally, the developed sensor system was fixed to the skin of a test person, and an arterialization time of 181 min could be determined.

## 1. Introduction

The respiration process encompasses the gas exchange of the two respiratory gases—oxygen (O_2_) uptake and carbon dioxide (CO_2_) elimination—on a cellular level, which diffuse between the alveoli and the blood in the pulmonary capillaries of the lung. The respiratory drive involves the synchronization of the central neural control (neural drive), the sensory input systems, respiratory muscles, and lungs [1]. A sufficient transport of O_2_ in the blood requires the protein hemoglobin to which O_2_ can be bound and transported dissolved in blood plasma [2]. CO_2_, on the other hand, is partially transported dissolved in blood, some is bound to hemoglobin, and the majority (~85%) of CO_2_ in blood is transported as part of the bicarbonate buffer system [3,4]. Depending on the lung function and the performance of the heart, the concentration of both gases in the blood varies. The differences in partial pressures of the respiratory gases between two locales drive the diffusion process between air and blood in the lungs and between blood and respiring tissues in the body. The diffusion of CO_2_ across the alveolar–capillary membrane is more rapid than O_2_ diffusion due to its greater solubility [2]. Lung disease can have severe influences on the blood gas composition. The primary goal in the care of sick patients is to ensure an adequate O_2_ supply to the tissues and vital organs, which is highly relevant for neonates [5]. The amount of CO_2_ present in the blood depends on the partial pressure and the presence of oxyhemoglobin [2]. Under normal physiological conditions, the CO_2_ partial pressure (p_a_CO_2_) in arteries is about 40 to 46 mmHg [6]. Transcutaneous measuring device developers typically approximate 7.0 mmHg p_a_CO_2_ ≈ 1 vol. % CO_2_ gas concentration, which would estimate the normal CO_2_ concentration levels in the blood to be ~6 vol. % [7]. Mismatched ventilation and blood flow can cause severe damage if not monitored. A deviation from matching the inspired air and blood flow from the normal ratio of 1:1 causes either an overventilation or underventilation of the alveoli in relation to the blood flow [8]. Hypoxia, a condition of low O_2_ and high CO_2_ content in the tissue and not meeting the metabolic needs of the cells resulting from underventilation, is as dangerous to neonates as it is to any other patient group, although neonates may be more resistant to hypoxia. However, hyperoxia, a condition with a greater O_2_ and lower CO_2_ content of the tissues and organs occurring when tissue and organs are exposed to an excess supply of O_2_ (overventilation), is notably dangerous for neonates as exceeding a certain level could cause brain damage. This might occur during artificial high-frequency oscillatory ventilation. 

To monitor the O_2_ saturation of patients non-invasively, a pulse oximeter and analysis of the exhaled gas concentration are broadly used. The main drawback of these methods is the lack of reliability and accuracy compared to invasive blood gas analysis, which requires regular yet painful blood sampling. Alternatively, transcutaneous monitoring devices have been of interest for several decades as a non-invasive method. Locally heating the skin surface through the sensor electrode surface induces an increased blood flow, which results in a rise in respiratory gases diffusing through the skin surface and therefore giving an accurate representation of the arterial partial pressure of the respective gases [5]. Measuring O_2_ on the skin requires sensor operation temperatures >45 °C to be able to detect O_2_ that diffuses out of the skin, which might cause skin irritation and painful burn injuries. Alternatively, CO_2_ starts diffusing out of the skin surface at a sensor surface temperature of only 37 °C, which is a more comfortable temperature for patients and infants [5]. The main benefits of transcutaneous CO_2_ blood gas concentration determination on the skin of patients in comparison to measuring the exhaled CO_2_ concentrations are the negligible influences of the patient’s age, body temperature, and lung diseases [9,10,11,12,13,14].

To date, transcutaneous p_a_CO_2_ monitoring devices for intensive care units are based on sensor heads with pH electrodes. The main disadvantage of such electrochemical transcutaneous sensors are drift phenomena that require a recalibration almost every 12 h [15]. To calibrate the sensors, a gas cylinder needs to be integrated into the complete measurement system, increasing the size and complicating the handling of the whole system, which restricts the implementation possibilities (e.g., mobile usage in ambulances or in overcrowded hospitals). A second calibration option includes replacing the sensing electrode and recalibrating the sensing device externally. Both approaches involve very time-consuming operational sequences, representing an additional burden for the nursing staff. Because of this, alternative detection technology requiring less maintenance is highly desirable.

Metal oxide resistive CO_2_ sensors, which change their resistance when in contact with the target gas, can be miniaturized and produced in large batch numbers through basic micro-electro-mechanical system (MEMS) manufacturing processes. Most current metal oxide gas sensors work at high temperature ranges (200–450 °C), which increases the sensor’s total energy consumption and with that reduces the sensor’s service life [16]. The doping of the sensitive materials needs to be reproduced reliably, which is critical for medical devices. It would require expensive quality and process control methods, as well as expensive high-precision manufacturing tools.

Cascales et al. present a wearable transcutaneous CO_2_ sensor that uses pH-sensitive fluorescent dyes [17]. Different fluorescent intensities indicate exposures to different CO_2_ concentrations. The fluorescent dyes are embedded into a hydrophobic polymer matrix. This detection method requires frequent changes of the sensitive film, as the fluorescent material experiences bleaching within a few hours. Furthermore, the polymer matrix might experience degradation over time caused by the used excitation wavelengths of the LEDs or anti-viral liquid disinfectants used in medical environments for hygienic reasons. In addition, the synthesis of these fluorescent dyes is a complex multi-step process where every step represents a potential source of errors or minor unintentional variations that might influence the reproducibility of different dye batches.

The photoacoustic (PA) detection method requires an acoustic transducer (e.g., MEMS microphones), which can be produced on a large scale, a light-emitting diode (LED) with an emission wavelength at which the target gas absorbs the light, and computerized numerical control (CNC) machined mechanical components, which can be reproduced with high reliability and accuracy [18]. In contrast to the previously mentioned detection methods, a PA sensor is an optical and much more stable alternative with minor maintenance required during operation times exceeding several days and up to several weeks. Furthermore, it does not rely on gas-sensitive chemical films or materials that demand complex production lines and might cause signal instabilities after short operation periods of a few hours. MEMS microphones are commercially available and can be operated for many years with no detectable ageing signs, as they are being used extensively in the mass market of telecommunication devices. In addition, commercially available high-power LEDs currently operate continuously and reliably over long time periods of several months while consuming low energy levels [19]. PA sensors have been mainly used as indoor air quality sensors and are mostly executed as one-chamber PA systems, which shows high cross-sensitivities to humidity and other present gases. An alternative and significantly more stable approach is the two-chamber PA detection method used in this work. 

We present a miniaturized non-dispersive infrared (NDIR)-based PA sensor system with an outer diameter of 18.5 mm and a total height of 11.5 mm, which is applied for the first time as a transcutaneous sensor on a test person’s skin. The continuous, reliable, and long-term stable CO_2_ detection of the developed two-chamber-based PA sensor has the potential to replace electrochemical transcutaneous sensors, as we were able to miniaturize the whole sensor system, including the analysis electronics, to a comparably small size. In addition, the newly developed PA sensor system was validated during laboratory measurements and compared to a commercially available CO_2_ NDIR air quality sensor. 

## 2. Theoretical Background of the Photoacoustic Sensor System

Very often, CO_2_ sensors are based on NDIR technology, equipped with an infrared light emitter (e.g., an LED or MEMS heater) and an infrared light detector (e.g., a pyroelectric detector or thermopile), and make use of the light-absorbing property of CO_2_ in the mid-infrared spectral region. NDIR sensors measure the target gas concentration based on the light intensity reaching the light detector, and the Beer–Lambert law can be assumed as a valid approximation of the light attenuation [20]. The two major drawbacks of NDIR sensors are the water and multi-gas interference and the high detection limit [21]. This is partially overcome, in particular for CO_2_ detection, by using optical filters and interference correction factors [22]. An improvement in the accuracy and sensitivity of the sensor were achieved by using novel infrared sources like micro-hotplates or LEDs and detectors like microbolometers to reduce the response time and sensitivity [23,24,25]. Furthermore, improving the optical designs by using a reference cell which overcomes issues occurring due to overlapping spectral absorbance bands of interfering gases and the target gases as well as maintaining the sensor stability influenced by the infrared (IR) source and detector aging had a positive impact on the sensor performance [26]. Another design approach uses the absorption properties of the target gas itself as the filter medium by combining the target gas with a sound transducer—in this case a chamber-separating membrane capacitor—into one hermetically sealed chamber. This principle was already realized in the “Ultra-Rot-Absorptions-Schreiber” (URAS), an infrared photoacoustic detection system for gases [27]. In addition to the separate measurement and reference absorption paths, two detection chambers are filled with the target gas and are separated by a membrane that transforms the pressure differences between both detection chambers which are caused by the varying sound wave strengths generated in each detection chamber and are related to the amount of absorbed light by the target molecules in the absorption path.

Improving the LED power has been the focus of many commercial LED developers over the past decade, which made high-power LEDs a convenient light source for low-cost PA systems, replacing thermal emitters due to the narrow band of IR, faster stabilization and modulation times, low power consumption, and low cost [22,25,28]. As shown in [29], a commercially available mid-infrared (MIR) LED can be modulated electrically at a modulation frequency of up to several hundred Hz while enabling reliable PA gas detection, which is not the case when using comparably slow thermal IR emitters. The used MEMS microphones are optimized to higher frequency ranges, which favors the use of LEDs. Higher modulation frequencies reduce the influence of surrounding noise on the measured acoustic signal.

In [30], the realization of a miniaturized PA methane gas sensor based on the differential Helmholtz resonator principle was presented using two commercial MEMS microphones to detect the differential PA signal. In [31], it was shown that a PA NDIR setup of an LED-based CO_2_ sensor without a reference channel and using a MEMS microphone that is placed into a CO_2_-filled detection chamber is feasible and long-term stable.

The sensitivity of a PA gas sensor is mainly dependent on the design of the PA cell [18]. In the case of a non-resonant technical configuration of the PA sensor, the modulation frequency of the light source is far below the lowest resonance frequency of the measurement cell and, as a result, does not generate signal amplification. 

**Figure 1:**
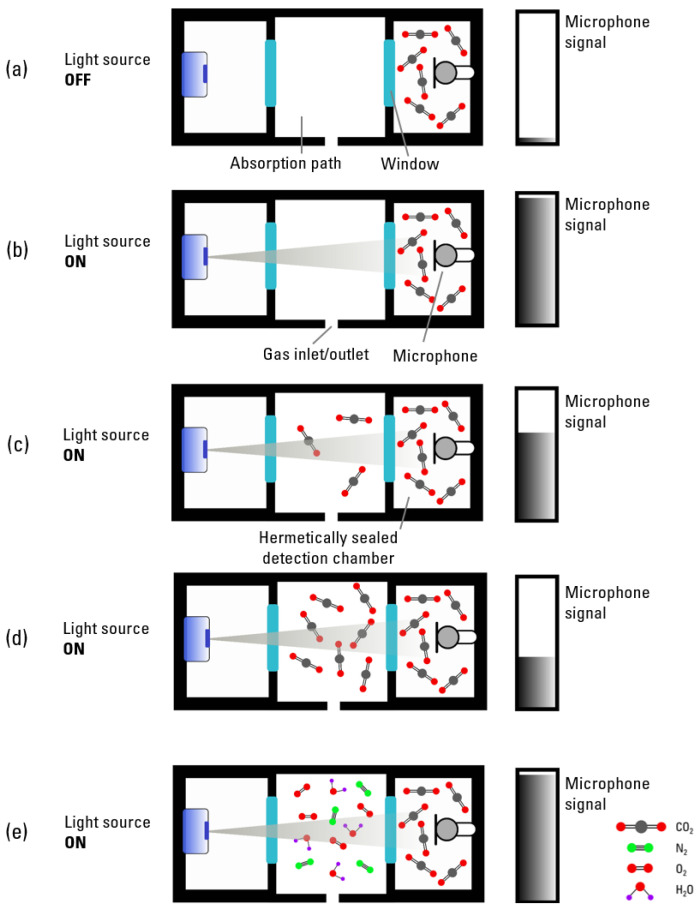
Theoretical concept of a two-chamber-based photoacoustic (PA) CO_2_ sensor. (**a**) Minimal microphone signal as light source is off. (**b**) Maximum microphone signal as light source is turned on and absorption path is not filled with the target gas, which results in the highest PA signal generation in the detection chamber. (**c**) Slightly reduced microphone signal—when compared to (**b**)—as the target gas (CO_2_) in the absorption path absorbs the light partially, resulting in a weaker acoustic wave generation in the detection chamber. (**d**) With increasing CO_2_ concentration in the absorption path, the microphone signal decreases further, which is in direct relation to the target gas concentration. (**e**) Maximum microphone signal detected without CO_2_ in the absorption chamber.

Our two-chamber PA sensor system consists of a hermetically sealed detection chamber containing the microphone and 100 vol. % CO_2_, a measurement cell (absorption path) that is placed between the light source and the detection chamber, and an LED with a defined emission wavelength. The measurement cell is filled with the gas mixture that is required to be analyzed. When the measured gas mixture contains the target gas, the microphone signal is reduced, as the target gas molecules in the measurement cell absorb a part of the energy, causing less light to reach the detection chamber, resulting in a weakened acoustic signal generation (Figure 1). The change in the microphone signal directly depends on the target gas concentration in the tested gas matrix, enabling its quantification. Filling a carrier gas mixture into the measurement cell will theoretically not lead to a significant change in the detected PA signal, as shown in Figure 1e, as no light is absorbed in the absorption path. In this case, the maximal light intensity reaches the detection chamber, which results in a maximum PA signal generation. A lock-in amplifier performs a multiplication of an incoming input signal, in our case the microphone signal, with a reference signal, in our case the modulation frequency of the light source [32]. It then applies an adjustable low-pass filter to the result, which is called demodulation. During this process, the signal at the frequency of interest—the modulation frequency—is isolated from all other frequency components.

The sensor response as well as the reference signal are used by the lock-in amplifier to determine the amplitude and phase of the detected signal, which is achieved through a dual-phase demodulation circuit. The input signal is split and separately multiplied with the reference signal and a 90° phase-shifted copy of it. The two generated output signals then pass through configurable low-pass filters, which results in two separate outputs, *X* and *Y*. The amplitude *R* and the phase *θ* can then be derived from *X* and *Y* by a transformation from Cartesian coordinates into polar coordinates using the following equations:

(1) $$R=\sqrt{X^{2}+Y^{2}},$$

and

(2) $$\theta =arctan\left(Y,X\right).$$

## 3. Material and Methods

### 3.1. Laboratory Setup

**Figure 2:**
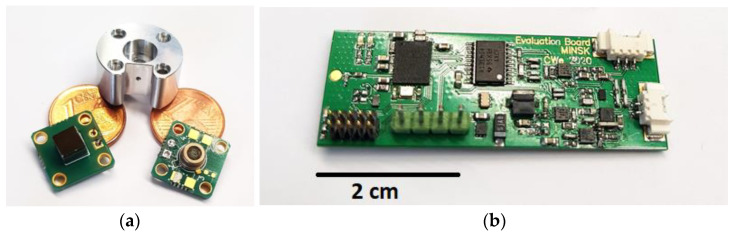
The components of the laboratory setup of the sensor system. (**a**) From left to right: PA detection chamber mounted to the readout printed circuit board (PCB), aluminum measurement cell including mounting holes, and 4.3 µm LED with Ф_e_ = 1 mW mounted to a PCB. (**b**) Evaluation board of the PA sensor system laboratory setup, in which the lock-in amplifier represents the core component.

We developed a laboratory setup of the PA CO_2_ gas sensor system, at first with a large version of the optical sensor and signal analysis electronics (Figure 2). All measurement cells were manufactured from aluminum due to its very good optical properties in the IR region (~97% reflectivity at a wavelength of 4.3 µm). In addition, the easy mechanical processing of aluminum yields suitable surface properties, which are essential for optical sensors. The volume of the cylindrical measurement cell was varied by changing the diameter (2.0, 2.5, 3.0, and 4.0 mm) while keeping the length constant at 2.0 mm and by changing the length (1.0, 1.5, and 2.0 mm) at a constant diameter value of 3.0 mm, as well as with 1 mm length variations of 2.0, 3.0, and 4.0 mm at a constant diameter value of 4.0 mm (see Table 1 for an overview of the different variations). 

We used the LED L15895-0430M (Hamamatsu, Shizuoka, Japan) with an optical output power of Ф_e_ = 1 mW as the light source and the MEMS microphone SPV1840LR5H-B (Knowles, Itasca, IL, USA) as the acoustic transducer of our sensor. The detection chamber was manufactured from steel. A layer of nickel and subsequently a thin gold layer were deposited on the steel surface of the detection chamber. The machining of the detection chambers and the coating depositions on the steel surface were carried out by Simek GmbH (Ilmenau, Germany). Afterwards, the microphone was placed inside the chamber and electrically connected using built-in glass feedthroughs. A metalized silicon (Si) window (t_Si_ = 500 µm) with a 500 nm thick silicon nitride (Si_3_N_4_) anti-reflection coating was soldered to the detection chamber in a 100 vol. % CO_2_ atmosphere. This grants a gas-tight sealing of the detection chamber, which is crucial for the long-term stability of the PA sensor.

### 3.2. Miniaturized Transcutaneous CO_2_ Sensor System Realization

**Figure 3:**
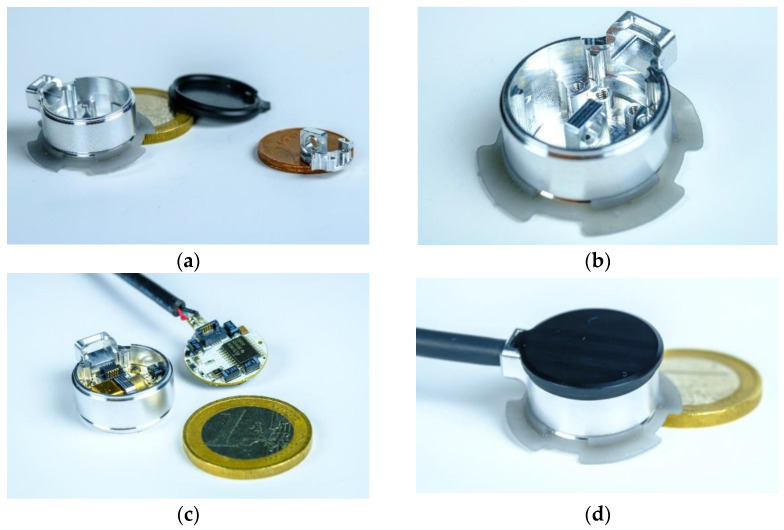
Miniaturized PA transcutaneous CO_2_ sensor system. (**a**) Aluminum sensor housing including a 3D-printed skin fixation ring, the polyoxymethylene (POM) lid, and the aluminum measurement cell (absorption path). (**b**) Measurement cell integrated into the housing and mechanically positioned through a specifically designed key–lock principle. (**c**) Two miniaturized PCBs comprising the analysis electronics of the sensor system and read-out PCB with the 4.3 µm LED and PA detector placed on opposite sides of the measurement cell. (**d**) Complete transcutaneous sensor system after integrating the PCBs and fixing the POM lid to the sensor housing.

**Figure 4:**
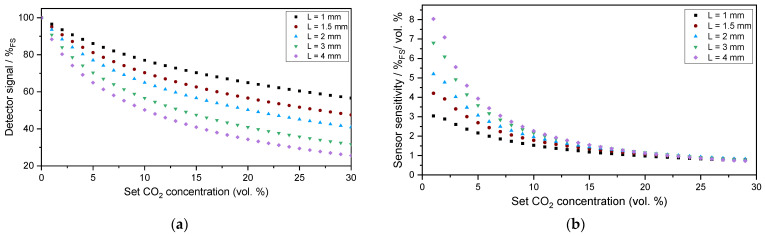
Simulations of the PA detector signal (**a**) and the first derivative of the PA detector signal (detector signal change per vol. % CO_2_) (**b**) for a measurement cell with a set diameter of 3.0 mm and lengths varying between 1.0 and 4.0 mm.

The outer diameter of the sensor housing is 18.5 mm, and the total height is 11.5 mm (Figure 3). The printed circuit boards (PCBs) comprising the analysis electronics and adapter board to control the LED power, which read out the detector signal and analyze the measurement signal through a lock-in amplifier, are designed to sizes suitable for integration into the miniaturized sensor housing. Furthermore, the measurement cell design of the PA sensor—also manufactured from aluminum—provides a mechanical coupling with the sensor housing and can be seen in Figure 3b. To maximize the optical performance of the sensor, the detector signal and sensitivity of the sensor were simulated. The diameter of the measurement cell was set to 3.0 mm to maximize the light throughput to the detection chamber, as the active area—the optical aperture—of the PA detector chamber is 3.00 × 3.00 mm^2^. A JavaScript software (version 1.8.5) was used to simulate the PA signal development depending on the CO_2_ concentration. The calculation is based on the emitted spectral power of the LED and the integral absorbed power in the detector, which was described further in [33] and uses spectroscopic parameters extracted from the HITRAN (high-resolution transmission molecular absorption) database [34]. The simulation results in Figure 4a show higher detector signal attenuations with increasing optical path lengths, which—at the same time—result in larger gas volumes and decrease the sensor reaction speed. The first derivation of the detector signal shown in Figure 4b, which corresponds to the detector signal over CO_2_ concentration changes (sensor sensitivity), shows a higher slope—with that a higher sensitivity—at larger lengths L and lower CO_2_ concentrations, while decreasing with narrower measurement cell lengths. Furthermore, an increase in the CO_2_ concentration shows a decrease in sensitivity while reaching a plateau at higher CO_2_ concentrations. 

Based on the simulation results, we decided to set the absorption path length to 2.0 mm, which is a trade-off between a faster sensor reaction due to a smaller total gas volume and a sufficient sensitivity of the PA measurement system. 

Due to the compact size of the sensor system, we designed a lid manufactured from the thermoplastic polyoxymethylene (POM) that can be applied to the housing without using screws, as shown in Figure 3d, which spares space and prevents electric shortages. The black cable exiting the sensor housing, which is shown in Figure 3c,d, can be connected to a USB port of a PC, and the generated data are logged using HTerm (version 0.8.5).

### 3.3. Methods

#### 3.3.1. Laboratory Setup

Laboratory measurements with CO_2_ using the different measurement cells of the developed laboratory setup were performed to investigate the influence of the length and diameter of the cells on the sensor signal. The CO_2_ concentration was varied between 0 vol. % and 18 vol. % in 3 vol. %, 2 vol. %, and 1 vol. % concentration steps using dry nitrogen (N_2_) as a carrier gas. The measurements were conducted using an LED current of 85 mA, the LED modulation frequency was set to 500 Hz, and the lock-in time constant—which corresponds to the lock-in integration time—was 1000 ms. 

Each measurement was conducted once for each measurement cell, recorded, and statistically analyzed. Each set gas concentration step was set to 5 min with a sampling rate of 1 s. For a proper mean value and standard deviation analysis of the laboratory setup signal behavior, we limited the analyzed data to 200 measurement points (n = 200) with the most stable signal of every set concentration step—corresponding to 200 s—per test gas composition. The total gas flow was set to 1.0 L/min throughout the measurements using the mass flow controller EL-FLOW Select F-121M (Bronkhorst High-Tech B.V., Ruurlo, The Netherlands) for all gases. The measured gas matrix concentration is set by varying the relations of the respective gas flow velocities. The average and standard deviation of the detected signal are presented in Section 4.

#### 3.3.2. Miniaturized Sensor System

The performance of the developed sensor system and a commercial NDIR CO_2_ sensor ExplorIR-W-20 (Gas Sensing Solutions Ltd., Glasgow, UK) was compared in simultaneous laboratory measurements using both sensors. The experiments comprised 1 vol. % CO_2_ concentration variations between 0 and 19 vol. % (dry N_2_ used as carrier gas) and evaluating cross-sensitivity effects of humidity (H_2_O) and oxygen (O_2_) on the detected signal, as these are the two potential main contributors of cross-sensitivities during transcutaneous applications in the medical environment. The O_2_ and H_2_O influences on the sensor signal were conducted at 8 vol. % CO_2_. The relative humidity (r.H.) was varied between 30 and 70% r.H. in 10% steps (10% r.H. corresponds to an absolute humidity of 5.68 g/m^3^_air_ at 42 °C and an air pressure of 1.0132 bar) through a gas matrix that comprised the set CO_2_ concentration, dry N_2_ as the carrier gas, and humidified N_2_. For the O_2_ concentration variations—5 vol. % variations in the range of 0 and 20 vol. % O_2_—dry N_2_ was the carrier gas, and the CO_2_ concentration was held constant at 8 vol. %. The measurements were conducted with test gases from the company Linde plc. (Dublin, Ireland). The purities of the used gases were ≥99.995% for CO_2_, ≥99.9999% for O_2_, and ≥99.9995 % for N_2_. For the humidity measurements, dry N_2_ is bypassed into a water-filled bubbler flask and is added to the dry test gas matrix. The humid N_2_ concentration is adjusted by a separate mass flow controller.

Throughout the conducted experiments, five measurement repetitions for each experiment were recorded and statistically analyzed. Each gas concentration step was set to 10 min with a sampling rate of one measurement every five seconds with the PA sensor and a default sampling rate of two measurements per second with the commercial ExplorIR-W sensor. For a proper analysis of the sensor’s behavior, we limited the analyzed data of the PA sensor to 100 measurement points (n = 100 × 5) and the ExplorIR-W sensor to 500 measurement points (n = 500 × 5) with the most stable behavior per test gas composition. The total gas flow was set to 250 mL/min throughout all measurements. The average and standard deviation of the detected signal were calculated and are presented in Section 4.2. 

During the measurements, the LED current amplitude of the PA sensor was set to 100 mA, while the modulation frequency was set to 500 Hz. Furthermore, the lock-in time constant was set to 1000 ms. The temperature of the photoacoustic sensor was kept constant at 42 °C through thermal equilibrium between the current-induced heat of the PCB and the surrounding surface, which is caused by the constant thermal conditions. This was realized by the thermal coupling of the PCB to the aluminum sensor housing after mounting them together, as the density of the PCB circuits was increased at direct contact points of the aluminum housing. 

#### 3.3.3. Transcutaneous Measurements on a Test Person

**Figure 5:**
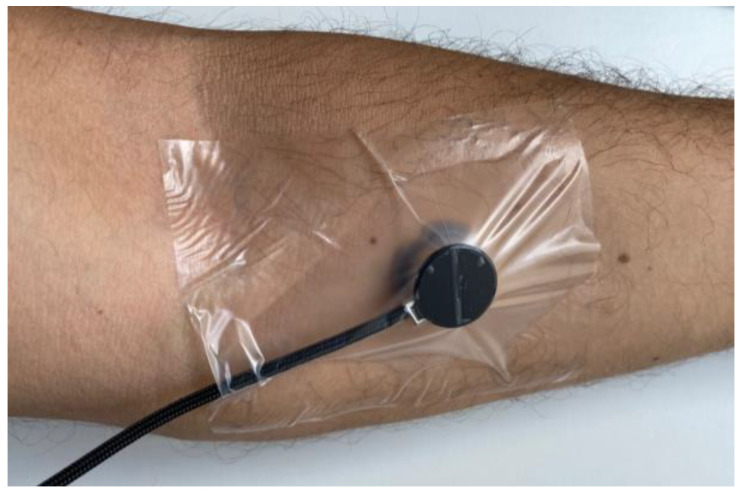
The miniaturized PA CO_2_ sensor was fixed to the left lower arm (flexor carpi radialis) of a test person using medical waterproof tape. To assure an air-tight skin–sensor interface, a contact gel from the company Sentec AG (Therwil, Switzerland) was applied to the skin surface at the desired measurement location before fixing the sensor to the skin.

To validate the functionality of the developed miniaturized sensor system during transcutaneous measurements, the CO_2_ sensor was fixed to the skin of a 32-year-old male test person without known pre-existing respiratory conditions or diseases. To assure an air-tight application of the sensor to the skin, which prevents the detection of interfering gases from the surroundings, a thin waterproof tape DracoFixiermull (DRACO) lightly pressed the flat sensor surface with the gas inlet to the skin surface (Figure 5). Furthermore, a gel-like liquid (contact gel) from Sentec AG (Therwil, Switzerland) was applied between the sensor and skin surface, which reduces influences through interfering surrounding gases and prevents skin irritations during transcutaneous measurements. The sensor was positioned and fixed to the left lower arm of the test person, specifically to the flexor carpi radialis, which is specified as a suitable area for transcutaneous CO_2_ gas measurements. During the measurements on the skin, the LED modulation frequency was set to 500 Hz at a current amplitude of 105 mA. Analogous to the previous laboratory measurements with the miniaturized sensor system, the lock-in time constant was set to 1000 ms, and the sensor housing temperature was regulated to 42 °C through thermal equilibrium between the current-induced heat and the skin surface. The sampling rate of the transcutaneous sensor measurement was one sample every five seconds.

The aim of this measurement was to determine the arterialization time of the developed sensor, which—in this case—comprises determining the time to fill the measurement cell volume of the sensor with the CO_2_ diffusing out of the skin.

## 4. Results

### 4.1. Laboratory Setup

**Figure 6:**
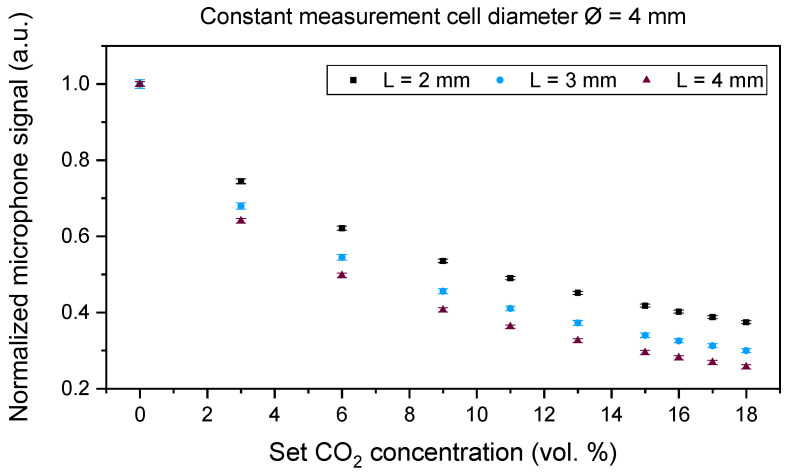
CO_2_ concentration dependency of the PA sensor signal at absorption path lengths L of 2.0 mm, 3.0 mm, and 4.0 mm. The diameter Ø of the absorption path was 4.0 mm.

**Figure 7:**
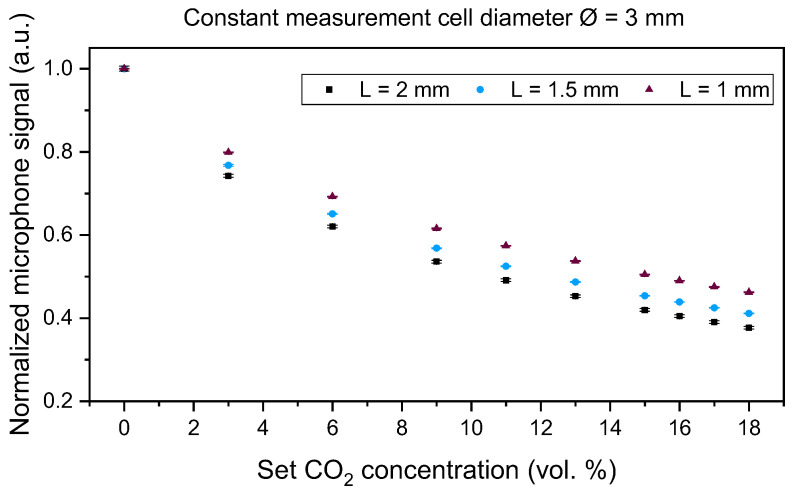
CO_2_ concentration dependency of the PA sensor signal at absorption path lengths L of 1.0 mm, 1.5 mm, and 2.0 mm. The diameter Ø of the absorption path was 3.0 mm.

**Figure 8:**
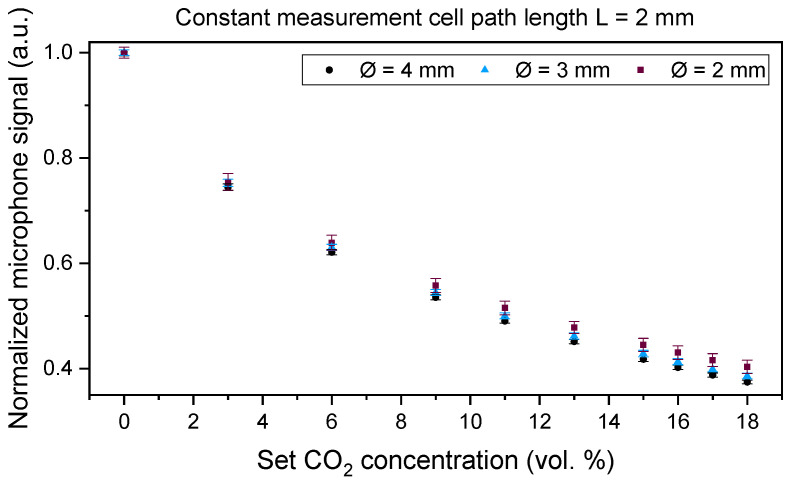
CO_2_ concentration dependency of the PA sensor signal at absorption path diameters Ø of 2.0 mm, 3.0 mm, and 4.0 mm. The absorption path length L was 2.0 mm.

A comparison of Figure 6, Figure 7 and Figure 8 shows a higher influence of the length of the measurement cell on the signal curve slope when compared to the influence of the diameter, which corresponds well with the simulation results in Figure 4 and the Beer–Lambert law, in which the path length L directly influences the absorbance while the diameter is not regarded in the respective absorption law. For simplicity in the discussion, we are regarding in the following the measured signal changes at an exposure to 6 vol. % CO_2_. In Figure 6 and when regarding the measured values at 6 vol. % CO_2_, a change in the absorption path length L from 2.0 to 3.0 mm resulted in a signal change of 12.1%, while a change in the measurement cell path length from 3.0 to 4.0 mm yielded a signal change of 8.8%. A 1.0 mm change in the diameter at a constant absorption path length L = 2.0 mm (Figure 8) yielded a signal change of 1.4% when comparing the measured values of the measurement cells with a diameter of 2.0 and 3.0 mm, while the comparison of a diameter of 3.0 and 4.0 mm results in a signal change of 1.6%.

The response time of the laboratory setup with the absorption path diameter = 3.0 mm and length = 2.0 mm was ~25 s, while the determined recovery time was ~26 s (see Supplementary Files). 

Furthermore, the conducted measurements showed that 1 vol. % CO_2_ concentration changes could be detected with all tested measurement cells of the laboratory setup, as can be seen in Figure 6, Figure 7 and Figure 8 where we varied the measurement cell length by 1.0 and 0.5 mm at constant diameter values of 4.0 and 3.0 mm (Figure 6 and Figure 7) and while varying the diameter in 1.0 mm steps at a constant measurement cell length (Figure 8). To find the optimal dimensions of the measurement cell, a trade-off between a faster sensor reaction due to the shortest possible absorption path length, i.e., a smaller measurement cell volume, and the largest possible diameter, i.e., large absorption cross section, for an increased sensor signal needs to be considered.

### 4.2. Miniaturized Sensor System

**Figure 9:**
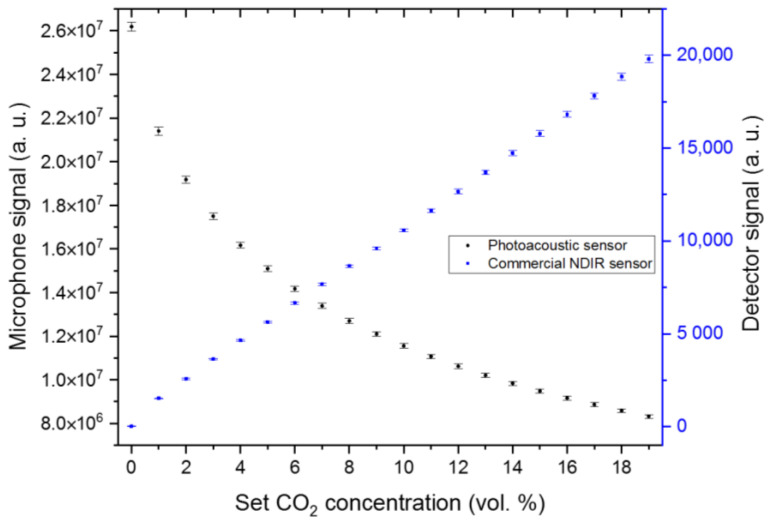
Comparison of the developed PA CO_2_ sensor with the commercial NDIR sensor ExplorIR-W (Gas Sensing Solutions Ltd., UK) at 1 vol. % concentration variations between 0 and 19 vol. % CO_2_. The tested gas matrix is a mixture of dry N_2_ and CO_2_. Each gas matrix variation was measured for 10 min.

Figure 9 shows the detected average signals of the miniaturized PA and commercial NDIR sensor at 1 vol. % CO_2_ concentration variations covering the concentration range of 0 to 19 vol. %, as this range is the maximum measurement range of the commercial NDIR sensor. The PA sensor can detect and differentiate all CO_2_ concentration changes in the tested measurement range. The detected microphone signal represents an exponential signal development, which corresponds well to the simulated signal behavior in Figure 3a. The average signal development of the commercial NDIR sensor shows a linear increase in the concentration range between 0 and 19 vol. %. The response time of the PA sensor was ~12 s, while the recovery time was ~10 s. The determined response and recovery times of the commercial NDIR sensor were about 84 and 80 s, respectively (see Supplementary Files).

**Figure 10:**
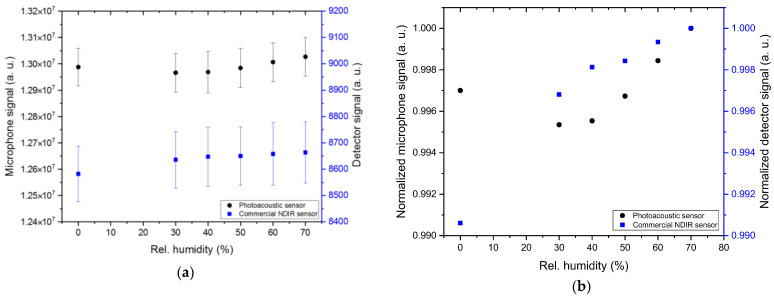
Comparison of the absolute (**a**) and normalized (**b**) signal values of the developed photoacoustic CO_2_ sensor with the commercial NDIR sensor ExplorIR-W (Gas Sensing Solutions Ltd., UK) at 10 vol. % concentration variations between 30 and 70 vol. % r.H. The tested gas matrix is a mixture of dry N_2_ and H_2_O at 8 vol. % CO_2_. Each gas matrix variation was measured for 10 min and repeated five times.

A comparison of the influence of H_2_O on the detected sensor signal of both sensors was conducted by quantifying the R^2^ of the linear fits of both sensor outputs along the tested humidity range, as the influence of H_2_O on the detected signals of both sensors is insignificantly low within the limits of the measurement accuracies (cf. Figure 10). The measurements were conducted at 8 vol. % CO_2_ while varying the relative humidity in 10% steps between 30 and 70 % r.H. The determined R^2^ of the photoacoustic sensor results was 0.34, while an R^2^ of 0.92 was calculated for the ExplorIR-W measurement results.

**Figure 11:**
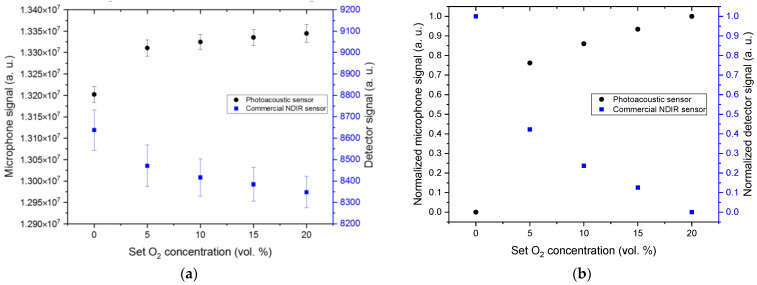
Comparison of the absolute (**a**) and normalized (**b**) signal values of the developed PA CO_2_ sensor with the commercial NDIR sensor ExplorIR-W (Gas Sensing Solutions Ltd., UK) at 5 vol. % concentration variations between 0 and 20 vol. % O_2_. The tested gas matrix is a mixture of dry N_2_ and O_2_ at 8 vol. % CO_2_. Each gas matrix variation was measured for 10 min and repeated five times.

For the next experiment, we regarded the O_2_ concentration range between 0 and 20 vol. % and varied the concentration in 5 vol. % steps at a constant CO_2_ concentration of 8 vol. % (cf. Figure 11). As can be seen in Figure 11, the signal of the ExplorIR-W detector signal decreases and the microphone signal of the PA sensor increases with increasing O_2_ concentration. 

### 4.3. Transcutaneous Measurement on a Test Person

**Figure 12:**
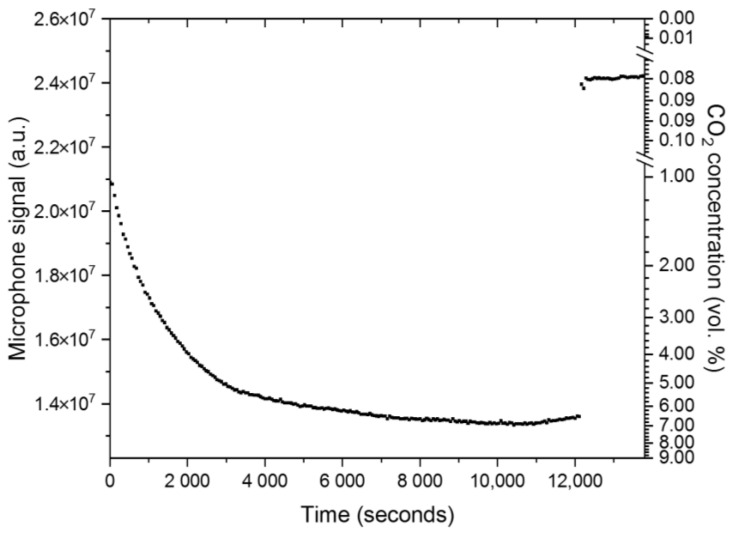
The slow detector signal change after fixing the miniaturized PA sensor to the skin surface of the test person. The decrease in the PA signal indicates the CO_2_ diffusing out of the skin surface and into the measurement cell of the CO_2_ sensor. The signal reaches its lowest measured value after 3 h (181 min), which represents the arterialization time of the developed sensor. After 202 min, the sensor is removed from the skin and the signal abruptly jumps to a value that corresponds to the surrounding CO_2_ concentration in air.

After applying the miniaturized PA sensor system to the skin surface and fixing it air-tight with the waterproof tape, the PA signal started to decrease, which indicates an instantaneous increase in the measured CO_2_ concentration. The detected CO_2_ concentration corresponds to the CO_2_ diffusing out of the skin surface and entering the gas inlet as well as the measurement cell of the tested sensor. The arterialization time of the sensor is identified as the time the sensor signal requires to reach its minimal value. In [15], the typical arterialization time range after applying a transcutaneous P_a_CO_2_ sensor on the skin is recommended to be 3–10 min, depending on the measurement site. As can be seen in Figure 12, the developed miniaturized sensor system requires around 181 min to reach arterialization time, which is significantly higher than the recommended and required range. Nonetheless, the primary tests on the skin showed the expected logarithmic signal decrease at the beginning of the measurement on the skin while reaching a minimum at a microphone signal corresponding to a concentration of about 6–7 vol. % CO_2_ diffusing out of the skin. Furthermore, the abrupt increase in the sensor signal after about 202 min reflects the expected signal change to the CO_2_ concentration of about 790 ppm within the office room caused by the removal of the sensor from the skin at that moment. The CO_2_ concentration in the office room was determined by the PA indoor air quality sensor SCD4x (Sensirion AG, Stäfa, Switzerland). The starting signal value at time t = 0 min varies from the measured value after removing the sensor (t > 202 min) because the data logging of the sensor signal was started prior to the secure skin fixation of the sensor, which required an additional 2 min during which CO_2_ already diffused out of the skin surface and into the applied PA sensor. The minimal signal fluctuations and the slight signal increase after reaching the signal minimum are most probably caused by unintentional arm movements of the test person, who had to keep his arm stretched out for three hours and the movement during that time to a minimum. Unintentional arm movements towards the end of the skin measurement might have caused minimal amounts of surrounding CO_2_ gas to reach the sensor. As the arterialization time of the developed PA sensor is too long, further and more detailed investigations are required after optimizing the inner gas volume of the PA sensor. These include reducing the total gas volume, which mainly consists of the gas inlet channel and the measurement cell, and conducting reference measurements with commercial transcutaneous CO_2_ sensor devices. 

## 5. Discussion

CO_2_ measurements with the laboratory setup included varying measurement cell dimensions like the length and the diameter. The measurements showed a dominating influence of the path length when compared to the influence of the measurement cell diameter. CO_2_ concentration steps as low as 1 vol. % were detected by the tested measurement cells. The miniaturized sensor system with an outer diameter of 18.5 mm and a total height of 11.5 mm was manufactured and compared to a commercial NDIR sensor through laboratory gas measurements. After completing simulations of the measurement cell by setting the diameter to 3.0 mm and varying the path length, the measurement cell was manufactured from aluminum with a total length of 2.0 mm. A comparison of the newly developed PA and a commercial NDIR CO_2_ sensor was performed through a series of different laboratory measurements, including CO_2_ concentration variations, and examining the influence of H_2_O and O_2_ on the sensor signal. The measurement results of the tested sensor systems showed that the PA sensor system and the commercial NDIR sensor were able to differentiate 1 vol. % CO_2_ concentration variations throughout the regarded concentration range. The determined response time of the miniaturized PA sensor was seven times and the recovery time eight times as fast as the commercial ExplorIR-W-20 sensor. Furthermore, the measurements showed that the influence of H_2_O on the sensor signal of both tested sensors can be neglected, yet both sensors showed a cross-sensitivity to O_2_, as with molecular collisions, O_2_ broadens the NDIR rotational spectral lines and influences the measured CO_2_ signals of both tested sensors [35]. Finally, the newly developed PA transcutaneous sensor was successfully tested on the lower arm of a test person, showing a significantly high arterialization time, which is mainly caused by the slow diffusion of the CO_2_ gas out of the skin. Based on the total gas volume (including the gas inlet channel and the absorption path dimensions) that needs to be filled with the target gas concentration and the 181 min the PA sensor requires to reach the signal minimum value, CO_2_ diffuses at a sensor surface temperature of 42 °C with approximately 2.1 µL/s out of the skin. As a consequence, a reduction in the total gas volume should be considered. Reducing the dimensions of the absorption path requires a detailed optical analysis to determine the influence of substantially small apertures on the PA signal quality and the sensor design, which will be considered during upcoming research. The measured 6–7 vol. % CO_2_ on the skin is within a realistic range of a healthy adult person, which is approximately considered to be ~6 vol. % and depends on the medical history and metabolism of each individual. 

## 6. Conclusions

A miniaturized PA sensor system and a laboratory setup—both based on the two-chamber PA concept—were developed and tested in a controlled gas laboratory environment. Comparing the miniaturized PA sensor for transcutaneous measurements to a commercial NDIR air quality sensor with similar measurement ranges showed that both sensors were able to differentiate 1 vol. % CO_2_ concentration variations, while less cross-sensitive signal influences on the PA sensor system to H_2_O and O_2_ were observed. In addition, the response and recovery times of the PA sensor system were significantly faster than the determined values of the commercial ExplorIR-W sensor.

The developed miniaturized PA sensor was successfully tested on the skin of a test person and consequently marks the first successful step towards a sensor for continuous and reliable p_a_CO_2_ determination through measuring the transcutaneous CO_2_ gas concentration.

## Figures and Tables

**Table 1:** Diameter and length variations (marked with “X”) of the evaluated measurement cells of the large laboratory setup of the photoacoustic sensor system.

Length L	Ø = 2.0 mm	Ø = 2.5 mm	Ø = 3.0 mm	Ø = 4.0 mm
*1.0 mm*			X	
*1.5 mm*			X	
*2.0 mm*	X	X	X	X
*3.0 mm*				X
*4.0 mm*				X

## Data Availability

Data underlying the results presented in this paper are not publicly available at this time but may be obtained from the authors upon reasonable request.

## Supplementary Materials

The following supporting information can be downloaded at https://www.mdpi.com/article/10.3390/s24020457/s1, Figure S1: The signal recovery time of the ExplorIR-W-20 sensor; Figure S2: The signal response time of the ExplorIR-W-20 sensor; Figure S3: The signal recovery time of the photoacoustic laboratory setup; Figure S4: The signal response time of the photoacoustic laboratory setup; Figure S5: The signal recovery time of the miniaturized photoacoustic sensor; Figure S6: The signal response time of the miniaturized photoacoustic sensor.
