# Supplementary figures and images for: Towards a Miniaturized Photoacoustic Sensor for Transcutaneous CO2 Monitoring
Source: Sensors (Basel). 2024 Jan 11;24(2):457. doi: 10.3390/s24020457 (PMC10820682; doi:10.3390/s24020457)

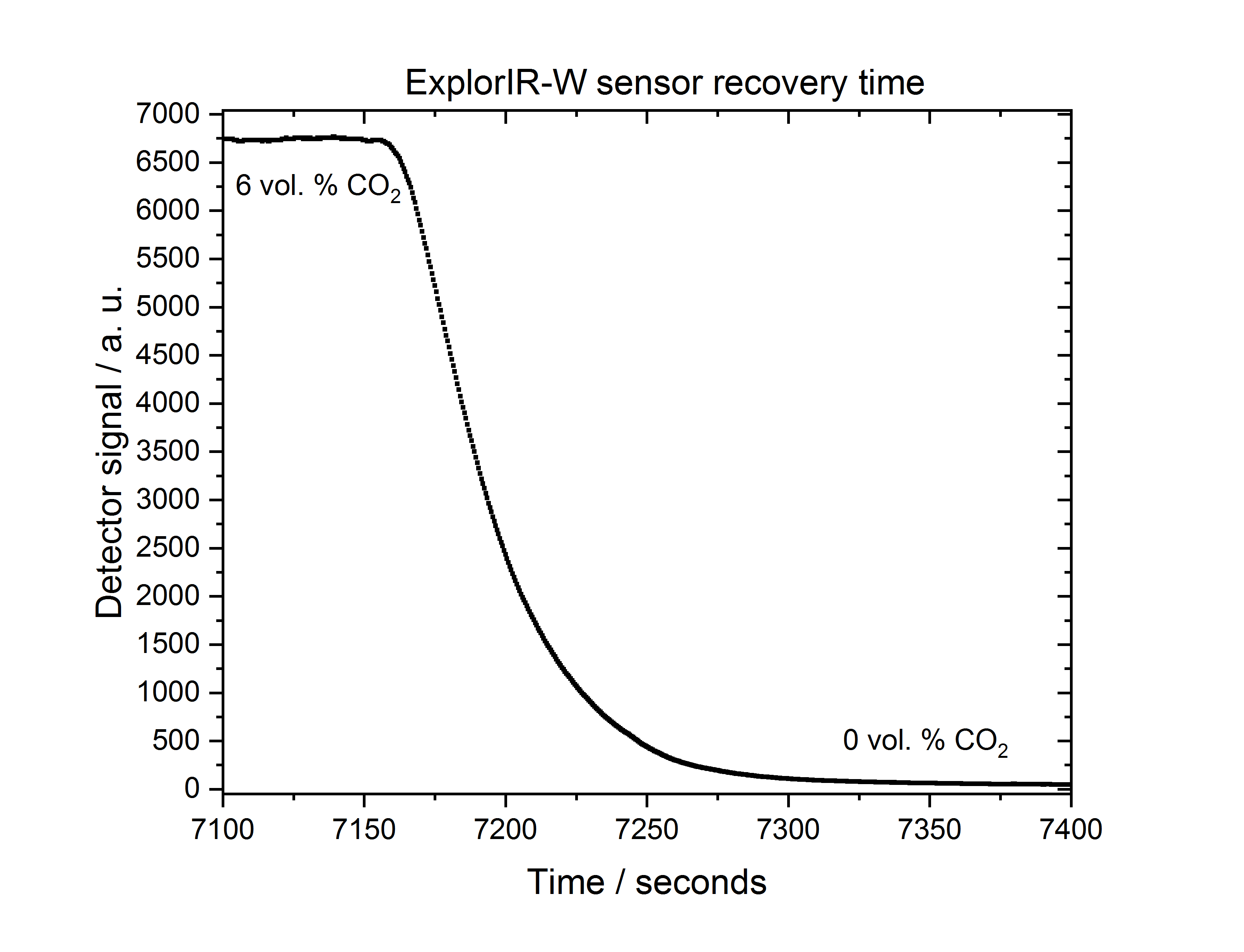

Supplement: Supplementary file 1 [file sensors-24-00457-s001.zip › S1_ExplorIR-W sensor recovery time.png]

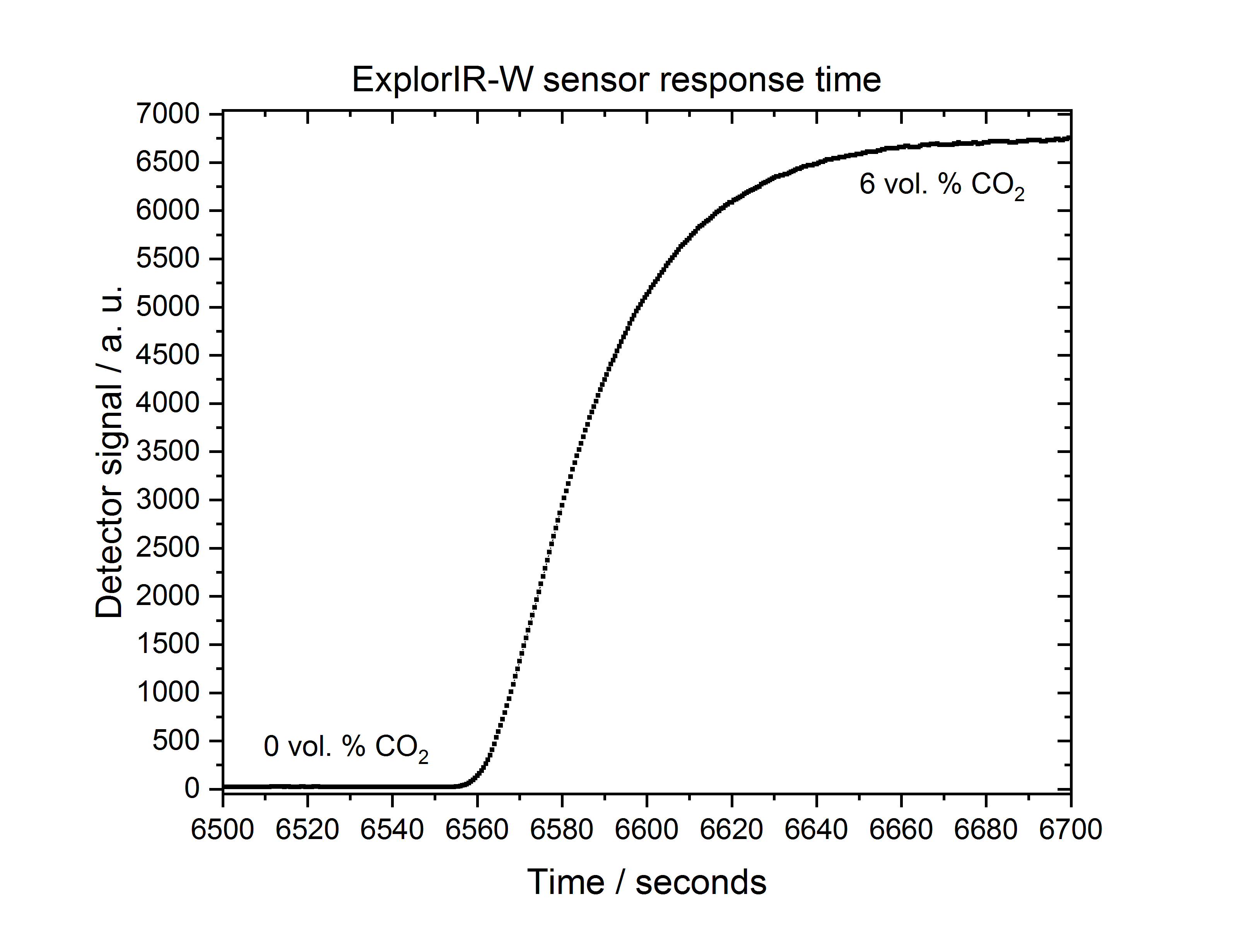

Supplement: Supplementary file 1 [file sensors-24-00457-s001.zip › S2_ExplorIR-W sensor response time.png]

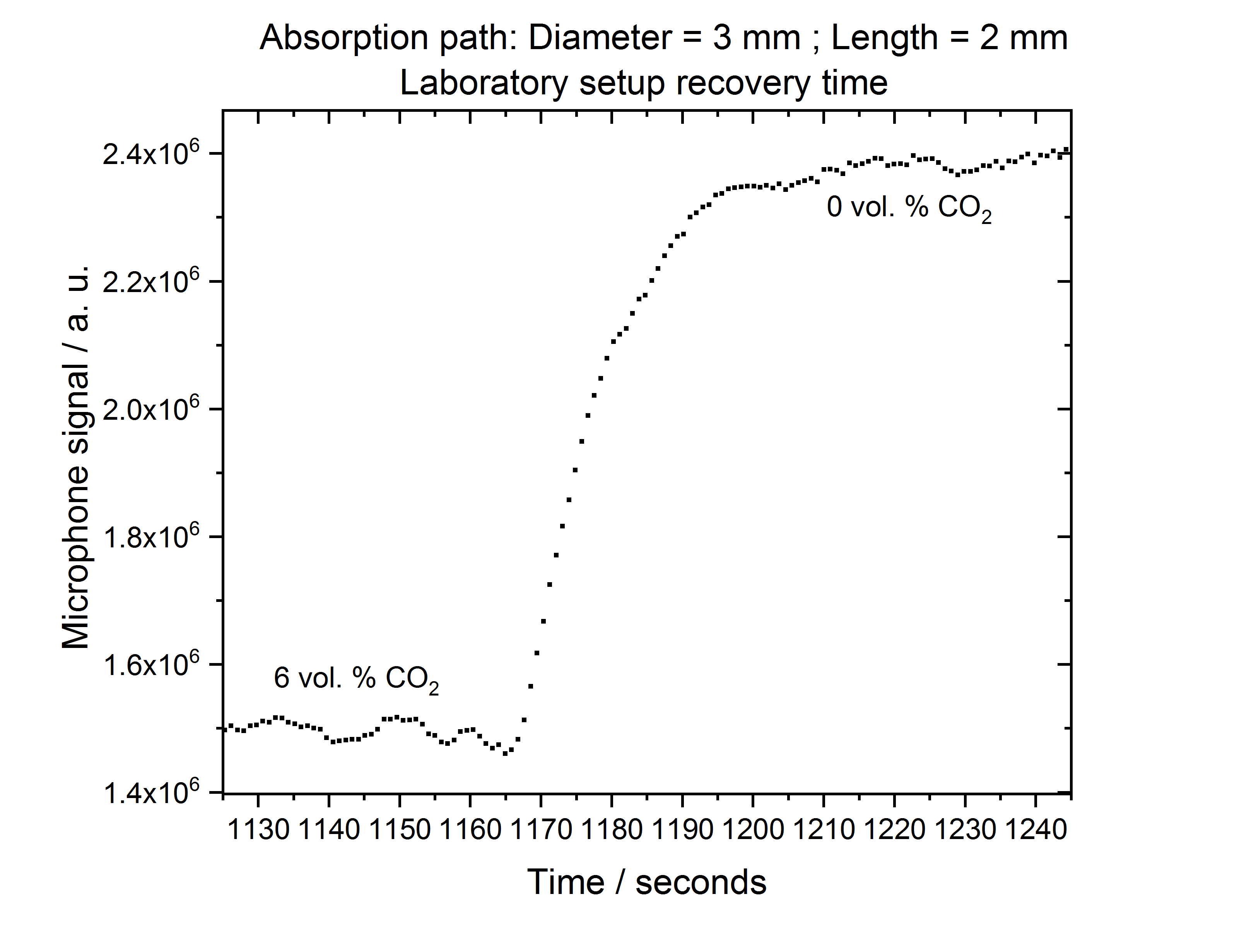

Supplement: Supplementary file 1 [file sensors-24-00457-s001.zip › S3_Laboratory setup recovery time.png]

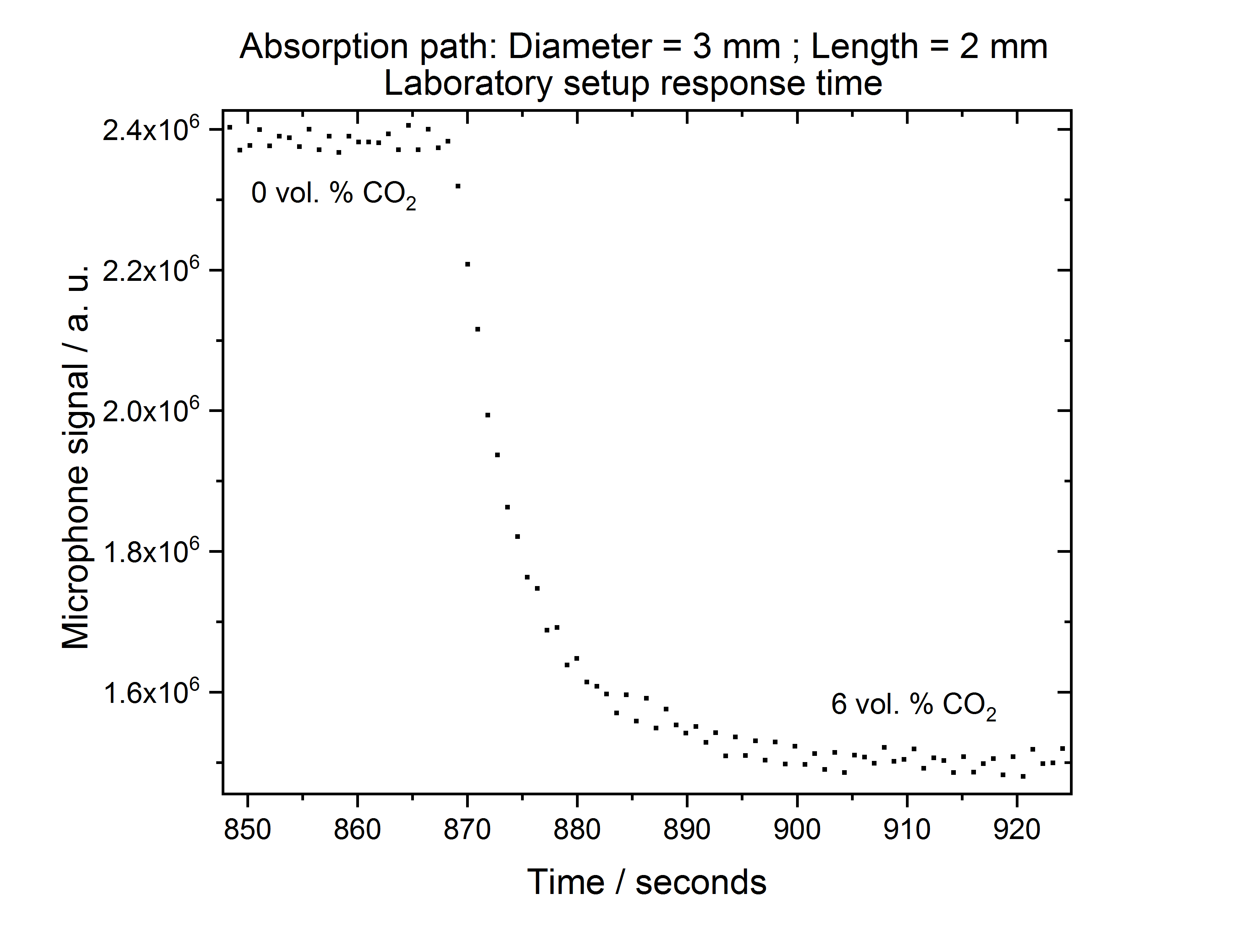

Supplement: Supplementary file 1 [file sensors-24-00457-s001.zip › S4_Laboratory setup response time.png]

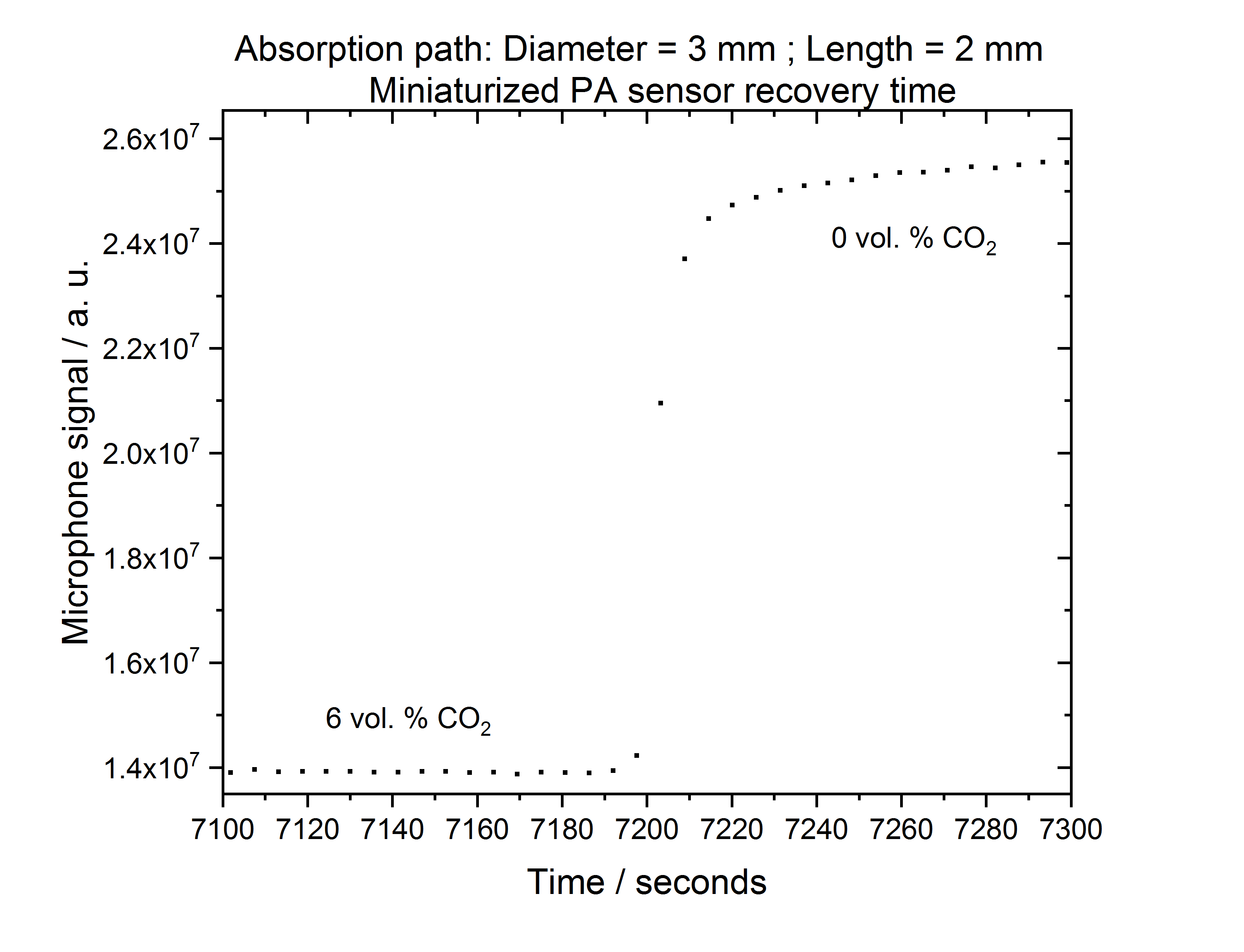

Supplement: Supplementary file 1 [file sensors-24-00457-s001.zip › S5_Miniaturized PA sensor recovery time.png]

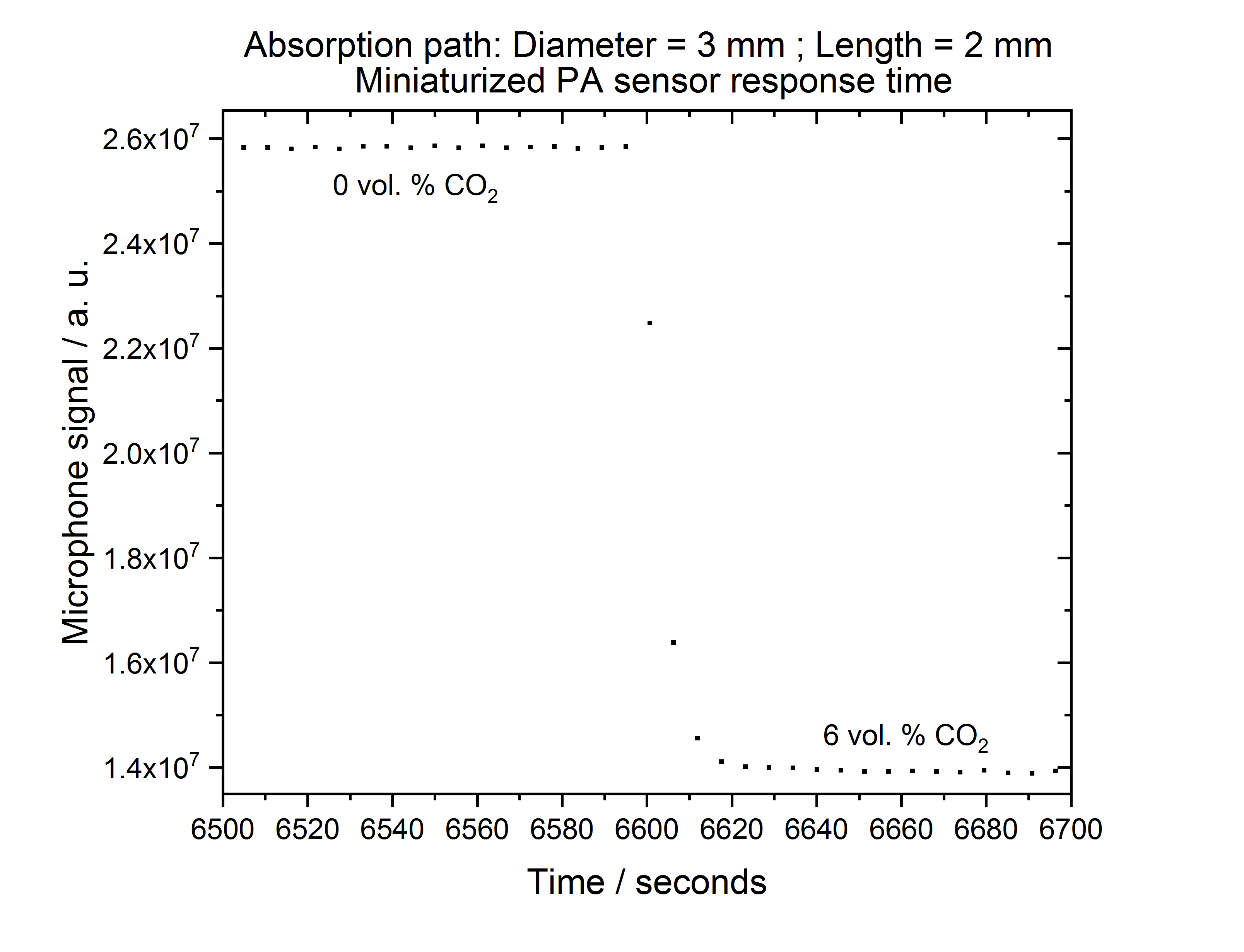

Supplement: Supplementary file 1 [file sensors-24-00457-s001.zip › S6_Miniaturized PA sensor response time.png]
